# Calibration and testing of a Raman hyperspectral imaging system to reveal powdered food adulteration

**DOI:** 10.1371/journal.pone.0195253

**Published:** 2018-04-30

**Authors:** Santosh Lohumi, Hoonsoo Lee, Moon S. Kim, Jianwei Qin, Lalit Mohan Kandpal, Hyungjin Bae, Anisur Rahman, Byoung-Kwan Cho

**Affiliations:** 1 Department of Biosystems Machinery Engineering, College of Agriculture and Life Science, Chungnam National University, 99 Daehak-ro, Yuseoung-gu, Daejeon, Korea; 2 Environmental Microbiology and Food Safety Laboratory, Agriculture Research Services, U.S. Department of Agriculture, Beltsville, United States of America; Uniwersytet Jagiellonski w Krakowie, POLAND

## Abstract

The potential adulteration of foodstuffs has led to increasing concern regarding food safety and security, in particular for powdered food products where cheap ground materials or hazardous chemicals can be added to increase the quantity of powder or to obtain the desired aesthetic quality. Due to the resulting potential health threat to consumers, the development of a fast, label-free, and non-invasive technique for the detection of adulteration over a wide range of food products is necessary. We therefore report the development of a rapid Raman hyperspectral imaging technique for the detection of food adulteration and for authenticity analysis. The Raman hyperspectral imaging system comprises of a custom designed laser illumination system, sensing module, and a software interface. Laser illumination system generates a 785 nm laser line of high power, and the Gaussian like intensity distribution of laser beam is shaped by incorporating an engineered diffuser. The sensing module utilize Rayleigh filters, imaging spectrometer, and detector for collection of the Raman scattering signals along the laser line. A custom-built software to acquire Raman hyperspectral images which also facilitate the real time visualization of Raman chemical images of scanned samples. The developed system was employed for the simultaneous detection of Sudan dye and Congo red dye adulteration in paprika powder, and benzoyl peroxide and alloxan monohydrate adulteration in wheat flour at six different concentrations (w/w) from 0.05 to 1%. The collected Raman imaging data of the adulterated samples were analyzed to visualize and detect the adulterant concentrations by generating a binary image for each individual adulterant material. The results obtained based on the Raman chemical images of adulterants showed a strong correlation (*R*>0.98) between added and pixel based calculated concentration of adulterant materials. This developed Raman imaging system thus, can be considered as a powerful analytical technique for the quality and authenticity analysis of food products.

## Introduction

Due to an increase in the number of food adulteration cases over the past decade, food safety has become a growing concern for both the food industry and for food safety organizations. In general, the major food commodities subjected to adulteration are spices, edible oils, honey, meat, and milk products [1]. Although all foodstuffs can potentially be subjected to adulteration, powdered food products are the most vulnerable because of the complex supply chain and a drift in nutritional and aesthetic quality over time. As such, ground material with a similar texture and color to the original powder can be added to increase quantity, while other compounds can be added to retain quality. To date, one of the most high profile examples of powder food adulteration motivated by economic gain was the addition of melamine to infant milk powder, where six children died and several thousand more were hospitalized [2]. In addition, the adulteration of paprika powder with coloring agents resulted in more than 60 hospitalizations [3].

Wheat flour is one of the main processed products of wheat and consumed worldwide. Therefore, the safety of wheat flour is of particular importance. However, several examples of wheat flour adulteration with hazardous chemicals such as benzoyl peroxide (BPO) and alloxan monohydrate have been documented in the literature [4–7]. Such adulteration is undesirable, as the addition of excessive quantities (higher than acceptance limit) of these compounds not only reduces the nutritional quality of the flour, but can also produce serious adverse health effects [7,8]. Although the maximum permitted concentration of BPO in food is 150 mg/kg, different countries follow different standards, and so a range of limits can be found in the literature [4]. However, no acceptable limit for alloxan monohydrate has yet been documented.

There is a long history of spice adulteration, particularly, paprika and chilli powders’ adulteration with various hazardous azo dyes such as; Sudan dye, Congo red, and Rhodamine B to obtain the desired aesthetic quality and make spices looks fresh [9,10]. The evidences of azo dyes adulteration in food stuffs and the detrimental effects of these chemicals to human health are well documented in literature [11]. Moreover, the adulteration of spices (particularly paprika and chili powders) with these synthetic coloring agents has become an increasingly serious issue in developing countries, particularly in markets where non-branded and loose spices are sold [12]. Therefore, the monitoring of different adulterant materials in powdered food products has become a crucial task.

Spectroscopic techniques, such as near-infrared (NIR), mid-infrared (MIR), and Raman spectroscopy have been widely used as fast and sensitive analytical techniques for the authenticity analysis of a variety of powdered food products [1]. However, these spectroscopic methods employ only a small portion of the sample for detection, and so the obtained spectra are often not representative of the whole sample, particularly if the sample is chemically heterogeneous, as is the case for adulterated materials. As an integrated alternative, hyperspectral imaging (HSI) techniques, such as NIR-HSI and Raman HIS, can provide a nondestructive means of screening food samples and have been used successfully for authenticity analysis of powdered food [13–16]. In general, Raman hyperspectral imaging (RHSI) produces narrower peaks than NIR-HSI, thereby producing more detailed information on a molecular level. Moreover, owing to its high specificity and sensitivity to various chemical components, RHSI can be proven more effective for detection of chemical adulterants in powdered food products.

The early applications of Raman imaging for food quality analysis were performed using a confocal point-scan Raman imaging microscopic system, which allowed the simultaneous analysis of various chemical components in carrot root [17], in addition to melamine screening in wheat flour, corn gluten, and soybean meal [18]. The majority of commercial Raman imaging systems are confocal, and so employ sub-centimeter-level detection, thereby limiting their use for the rapid analysis of samples exhibiting large surface areas. As such, the use of Raman imaging techniques for food quality and authenticity analysis must be fast and effective to allow the analysis of a large sampling area.

Recently, Qin et al. [19] developed a benchtop point-scanning Raman imaging system for food quality analysis, which was faster than existing microscopic-based Raman imaging systems. Despite being relatively fast, the sampling time was still rather long (typically a few hours), thereby limiting its use in the rapid screening of bulk samples bearing large surface areas. Therefore, in an effort to overcome the limitation of scanning time, Qin et al. [20] developed a line-scan Raman imaging system that realizes high-throughput macro-scale Raman imaging for food safety and quality research while maintaining spatial resolution and sensitivity. The developed system was further employed in a range of food quality and authenticity analysis applications [21]. Moreover, Wang et al. [22] used line-scan Raman imaging technique for detection and quantification of BPO adulterated to wheat flour. However, majority of studies utilizing Raman imaging technique for authenticity analysis of powdered food deal with only one type of adulterants; whereas, two or more adulterants can be added for the same purpose. Moreover, the commercial software used for Raman image collection of BPO adulterated wheat flour [22] and detection of other adulterants in powdered food [23,24] seems not to be applicable for effective and real-time visualization of Raman chemical image of adulterant materials which if possible, can further be used to make instant decision on food quality and thus, ultimately accelerate the speed of quality analysis tests in industrial environment.

To advance the application of Raman imaging in food quality and authenticity analysis, we herein report the design of a Raman imaging instrument similar to that developed by Qin et al. [20,21]. However, the main differences lie in optical assembly to insure uniform laser line excitation and the developed software for data collection and real-time visualization of Raman chemical images. In detail, the line-scan Raman imaging system developed by Qin et al. [20,21], utilize a scanning mirror, oscillating at a scan rate of 250 Hz (4 ms per full cycle sweep), and the laser point is spread at the vibrating mirror surface to form a divergent laser beam. Because the illumination system uses a vibrating mirror thus, mechanically sensitive. Therefore, to overcome from such constraint, in this study, a diode NIR laser system is customized which uses a unique arrangement of optics to generate a uniform laser line. Secondly, the developed system uses a user friendly custom-designed software which facilitate the real time visualization of Raman chemical images of adulterants and can further be extended for other food quality and safety application by incorporating the image processing algorithms, unlike the system based on commercial software, which limits users from adding image processing functions for specific applications.

Herein, more specifically we aim to explore the potential of the RHSI technique for the authenticity analysis of two different globally used powdered food products, in addition to developing custom-built software for the RHSI system to allow image acquisition and analysis by the addition of image processing algorithms for different applications. Furthermore, we wish to develop fast and effective Raman spectral and image processing algorithms for the visualization of concentration maps of adulterant materials present in food powders. Finally, we expect that the developed system will enable the fast and high-throughput screening of powdered food samples for authenticity analysis, while mapping a large sample area along the laser direction, which can be further expanded by increasing the sample-to-camera distance or by making some minor alterations to the developed system while considering the required spatial resolution for any particular application.

## Materials and methods

### The Raman hyperspectral imaging system

#### Instrument design

**Fig 1:**
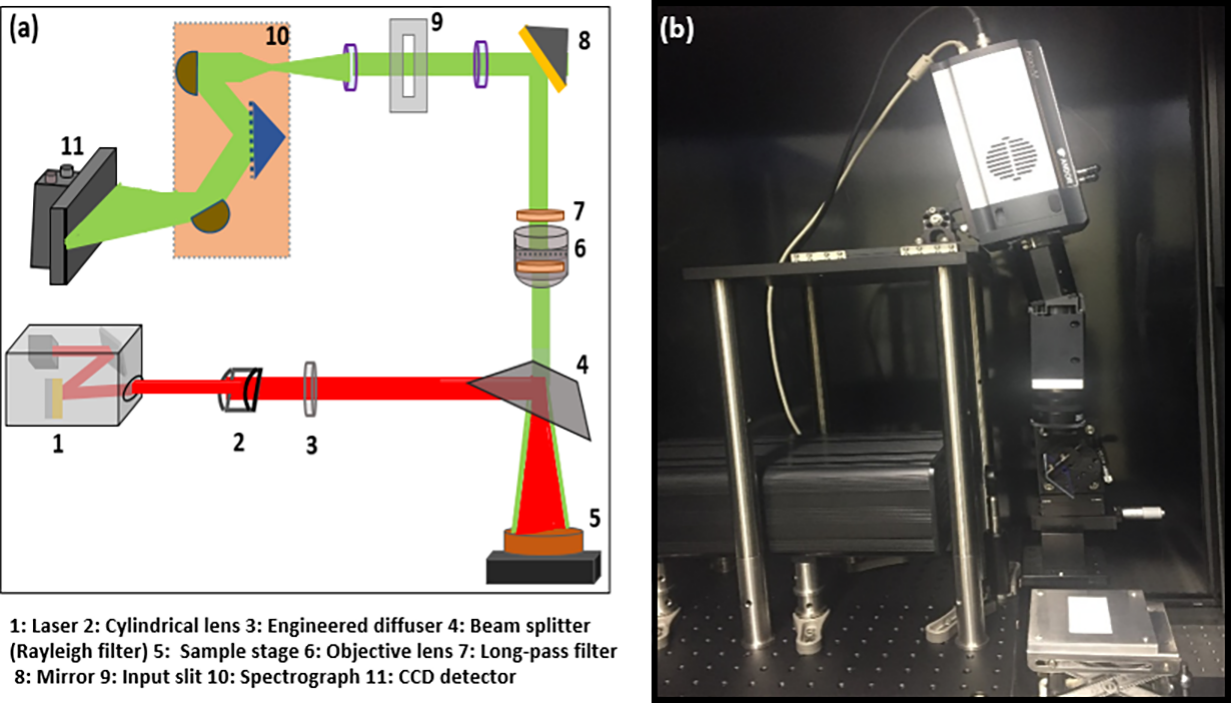
Optical design of line-scan RHSI system (The laser light to sample in red and scattered light from the sample in green color) (a), and photograph of a system used to acquire images from layered and mixed food powder samples (b).

The developed system consists of an optical assembly to ensure a uniform laser excitation and Raman signal collection, and was further coupled to a charge-coupled device (CCD) camera through the spectrometer to generate Raman images without interference from Rayleigh scattering. A schematic representation of the developed system is illustrated in Fig 1A and a photographic image of the system is shown in Fig 1B. A custom-designed diode NIR laser system (OptiGrate Corp. FL., USA) operating at 785 nm was used as the excitation source with a laser power of ~400 mW, which was measured at the surface of the sample using a digital power meter (PM100D, ThorLabs, Hans-Boeckler, Dachau, Germany). To generate a high-energy laser line, laser light from 19 emitters was mounted and focused onto a 785 nm bandpass filter. The filter was angled to reflect the filtered laser beam towards a reflection mirror, which was placed in such a manner that it reflected the laser beam out of the laser box without any loss in beam quality. The relatively small laser line was further expanded by passing through a cylindrical lens (*f* = 200 mm). However, the use of a cylindrical lens produces a poorly illuminated line, and is limited by the non-uniform and Gaussian-like intensity distribution [25]. An ideal laser line for Raman applications should be a well-defined line with a uniform width and intensity at a defined distance. A uniform line shape with a top-hat intensity beam profile and a homogeneous intensity distribution was therefore achieved using an engineered diffuser (ED1-L4100; ThorLabs, Hans-Boeckler, Dachau, Germany). However, the loss in beam intensity was obvious upon the application of the engineered diffuser as a result of overspill.

The laser line was then projected onto a 785 nm dichroic beamsplitter (Semrock, Rochester, NY, USA) and its path changed to a vertical orientation. The dichroic beam splitter (10 cm × 3.5 cm) was mounted on an xyz translation stage incident at 45° to reflect the laser beam to the image plane. The dichroic beamsplitter should reflect the majority of incident light while blocking the non-informative Rayleigh scattering generated during Raman data collection. The uniformity of the laser line on the sample surface was measured by scanning a Teflon sheet at two different vertical distances (i.e., 16 and 24 cm) from the center of the beamsplitter, giving line uniformities of ~10 and ~14 cm, respectively. The sensing module to collect the generated Raman signals is identical to Raman imaging system described in [19]. A manual iris C-mount lens (Schneider Optics, Xenoplan 1.4/23 mm NY, USA) was attached to the imaging spectrograph for aperture and focus adjustment. The generated Raman signals were further filtered to remove Rayleigh-scattered photons using two 785 nm long-pass filters and subsequently enter the imaging spectrograph via a slit. A 16-bit high performance CCD camera (iKon-M 934, Andor Technology, South Windsor, Conn, USA) was placed at the focus plane of the spectrometer to collect the dispersed signals and to create a two-dimensional (2D) image. A Computer controlled stepper motor-based translation stage (Velmex, Model XN 10-0180-M02-21, NY, USA) was integrated to move the samples during line-scanning. All hardware was installed in an enclosed black box to omit ambient light.

#### Software design

**Fig 2:**
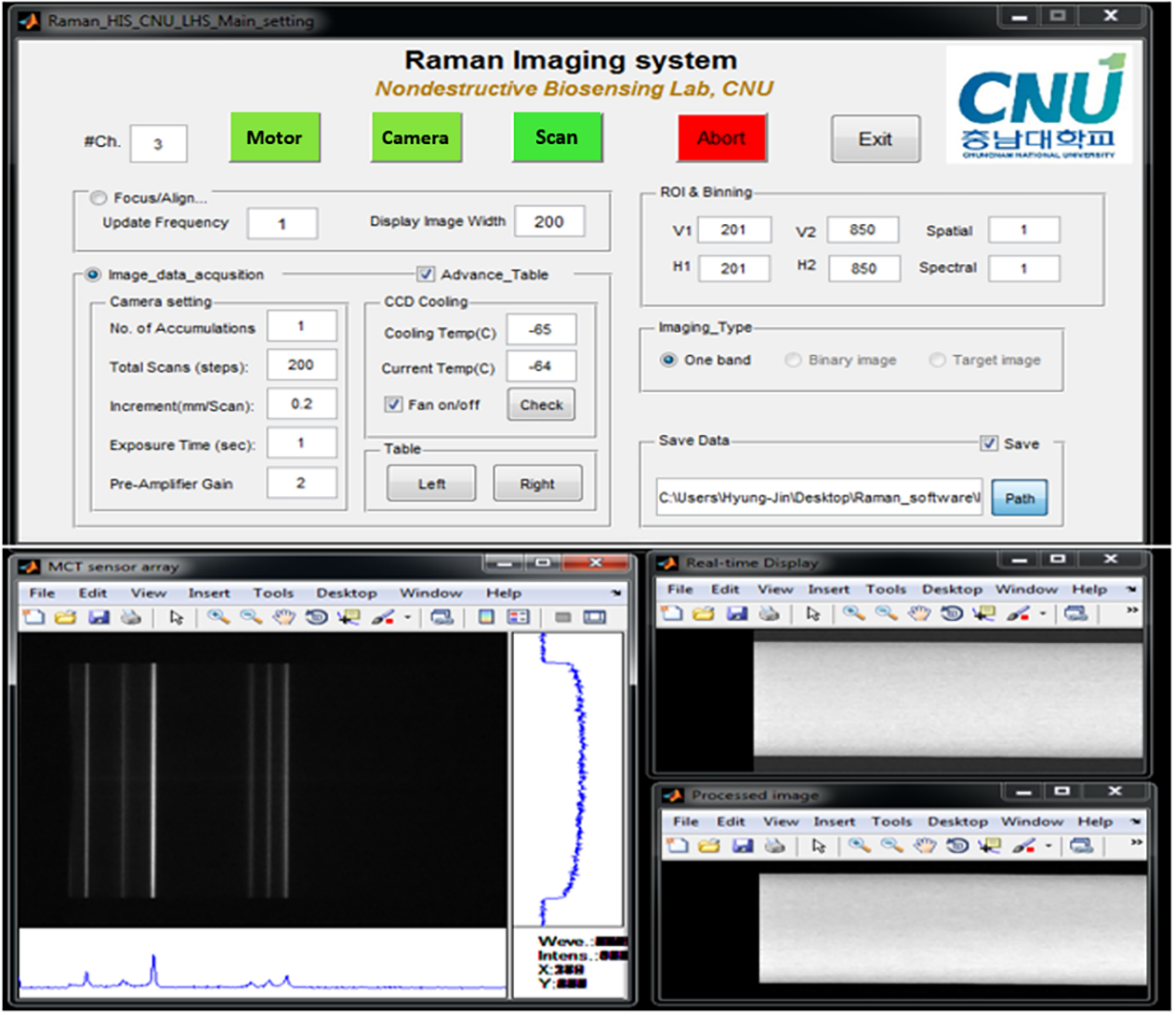
Software interface for image acquisition and system control.

Custom software for focus alignment, image acquisition, and RHSI system control was developed using MATLAB (version 7.14, Mathworks, Natick, MA, USA). This software also includes control of the RHSI camera and the sample stage. The display interface of the MATLAB code is shown in Fig 2. The Focus/Align panel was used to display the real-time image of the area array detector, and so can be used for adjustment of the focusing lens to obtain the optimal contrast prior to Raman data collection. The Image data acquisition panel provides details regarding the various input parameters required to control the camera and step motor for experimental data collection. This software scans the sample line by line, thus, generating a 2D spectral-spatial image of each step during sample movement, eventually allowing a 3D hypercube to be saved using a defined filename and saving path. The window on the bottom left of the figure shows the image of one scan line on the sensor array, where the horizontal direction is the spectral dimension and the vertical direction represents the spatial dimension. The snapshot of the software interface (Fig 2) was taken while measuring the Teflon sheet for laser uniformity analysis. A spatial profile and a spectrum extracted at the position of the mouse cursor allow Raman scattering to be monitored in both directions. In addition, the window on the bottom right of the figure shows the sample images during step-by-step scanning of the Teflon sheet, where the upper image represents a raw image of a set wavelength and the bottom image represents the processed (i.e., fluorescence corrected and median filtered) image of the same waveband. This system also includes other image processing algorithms, which enable the user to analyze and render the data in real-time. The developed software can easily be extended and customized for analysis of diverse samples integrating different data processing algorithms within the software.

#### System calibration

**Fig 3:**
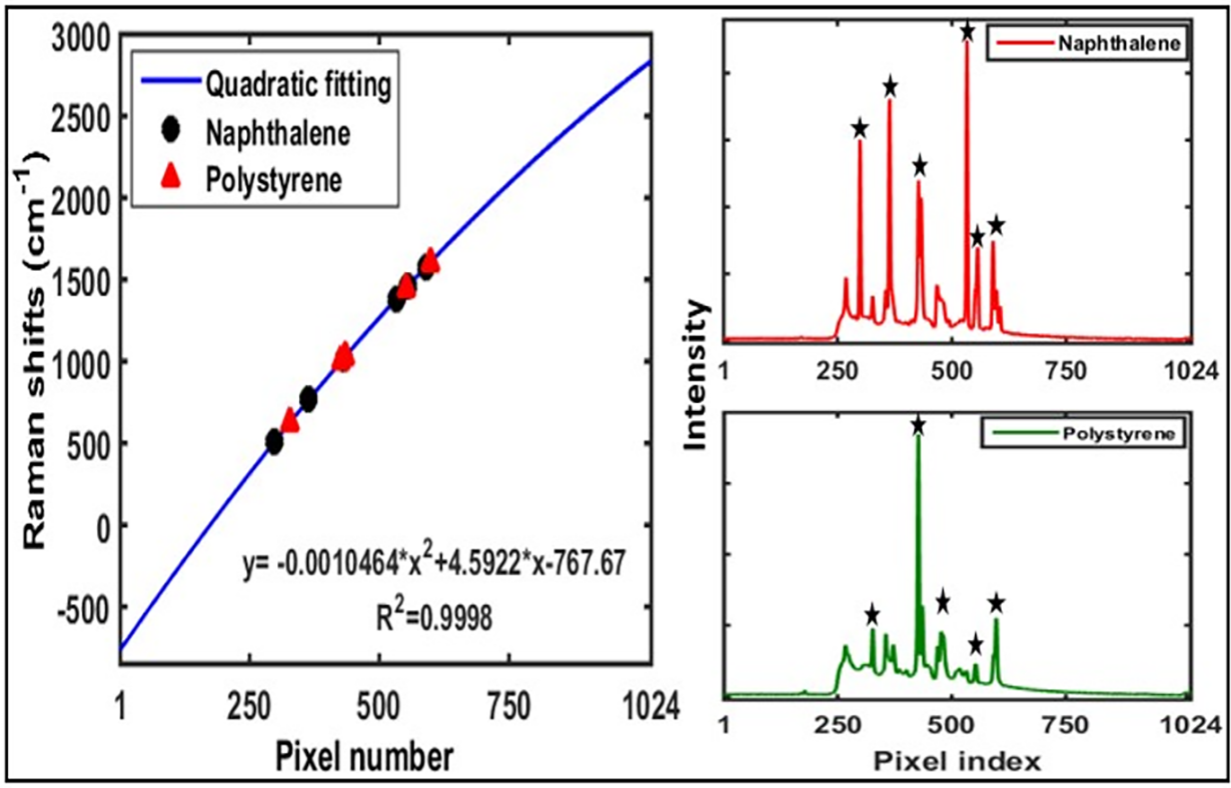
Spectral calibration for the Raman imaging system using a quadratic regression model.

Spectral calibration is a basic and routine procedure in all spectroscopic and spectral-imaging techniques. The goal is to assign a wavelength to all pixels along the spectral dimension of the CCD. In this study, the system was spectrally calibrate using two standard materials (naphthalene and polystyrene). As shown in Fig 3, six sharp peaks corresponding to naphthalene and five well-resolved peaks corresponding to polystyrene were employed to develop a quadratic regression to ultimately yield a regression equation by which the Raman shifts could be assigned to each pixel. The obtained results indicated that the system covers a spectral range from −763 to 2837.5 cm^−1^ (i.e., 740.6 to 1010 nm) and that it has an effective average spectral resolution of 3.5 cm^−1^. The spectral resolution at the full width at half maximum, which corresponds to the theoretical maximum resolution of the spectrograph, was tested according to the method reported in reference [20], and was determined to be ~15 cm^−1^. Spatial calibration was carried out using black and white striped paper. Under the desired settings, the calculated and measured image line lengths and spatial resolutions showed a perfect match.

#### Experimental sample preparation

**Fig 4:**
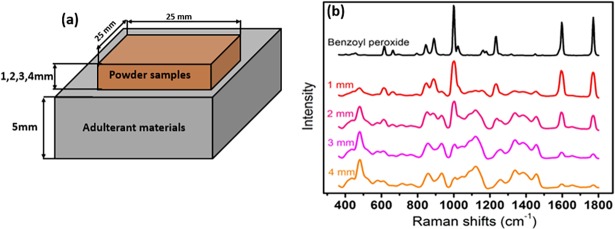
Schematic of sample holder for effective penetration depth determination of laser line through food powders (a), and mean Raman spectra of wheat flour of four different thickness over the adulterant material benzoyl peroxide (b).

Wheat flour and paprika powder samples were obtained from a local market in Korea, and four adulterant materials, namely BPO, alloxan monohydrate, Sudan-I, and Congo red dye (purity >95%) were purchased from Sigma-Aldrich (St. Louis, MO, USA). As quantification of the penetration depth of the laser line for the powdered food samples is important in food authentication applications to ensure all adulterant particles can be detected, the penetration depth was determined by placing the wheat flour and paprika powder samples at four different thicknesses over a 5 mm-thick layer of the adulterant material (Fig 4A). In this case, BPO was used for the wheat flour sample and Sudan dye was used for the paprika powder sample.

The powdered food samples were then adulterated at six different concentrations, where the concentrations were selected based on previous reports [4,26]. Thus, the required amount of each adulterant was added to the wheat flour (BPO and alloxan monohydrate) and paprika powder (Sudan dye and Congo red) samples to achieve target sample concentrations (w/w) of 0.05, 0.1, 0.25, 0.5, 0.75, and 1%. Thus, the 0.05% sample contained 0.05% of each adulterant and 99.9% of the pure powder (i.e., wheat flour or paprika powder). The samples were then mixed using a high-speed vortex mixer. Each mixture was prepared in duplicate, packed into an aluminum sample holder (80 mm × 40 mm × 2 mm), and the powder surface was leveled without pressure at the top edge of the sample holder using a spatula. The weight of each sample was selected to ensure that the majority of the sample could be scanned.

#### Raman data collection and analysis

Both the layered and mixed samples were scanned using the described RHSI system shown in Fig 1B. Raman images for both the layered and mixed samples were acquired using the same instrumental settings and parameters, with the exception of the total scanning line due to the small size of the layered samples. To increase the signal-to-noise ratio while maintaining a fast scan speed, three parameters were considered. More specifically, sample movement was set at 0.15 mm/scan through the conveyor unit to cover the spatial shape of sample, the exposure time was set to 1 sec, and the distance of the sample from the camera was set at 24 cm. At this distance, the length of the camera’s instantaneous field of view (IFOV) was approximately 150 mm ((slit length (14.2 mm) × measurement distance (240 mm))/lens focal length (23 mm) = 148.2 mm), and so the nominal pixel size along the laser line was 0.145 mm (148 mm/1024 pixels = 0.145 mm/pixel). Therefore, to obtain an almost square-shaped pixel and upon considering the particle size of the adulterant materials (i.e., 100–180 μm), the step size was set at 0.15 mm. At this setting, a total of 270 scans were collected to cover the whole sample length (40 mm) thus, the total scanning time for each sample was about 6 minutes. Prior to acquisition of the images, the laser light and camera were switched on for 30 min to stabilize the laser source and camera, and to improve the spatial uniformity and reduce noise. The samples were placed successively on the conveyor unit for step-by-step scanning under the camera FOV and the Raman images were acquired for the whole surface area of the packed powder samples for each concentration. The data were collected in the spectral range of 360–1800 cm^−1^ using a CCD spatial binning of 2. The major signals corresponding to the adulterant materials used in this study were mainly observed in the spectra between 360 and 1800 cm^−1^. To correct the raw images, dark current images were acquired with the laser switched off and the camera lens covered with its opaque cap.

The data acquired from Raman imaging instruments equipped with a CCD detector are usually characterized by occasional high intensity spikes, with fluorescence often being an issue. In this case, the collected Raman image data were first reorganized from a three-way array to a conventional two-way array (pixel × Raman shifts) and initially corrected by subtracting the dark current. To address the issue of unwanted fluorescence background signals, the adaptive iteratively reweighted Penalized Least Squares (airPLS) fluorescence correction method reported in literature [27] was applied to the Raman data. Moreover, a 2D median filter on every band image with a moving window of 3 × 3 pixels was applied to the fluorescence-corrected data to remove high intensity spikes [28].

**Fig 5:**
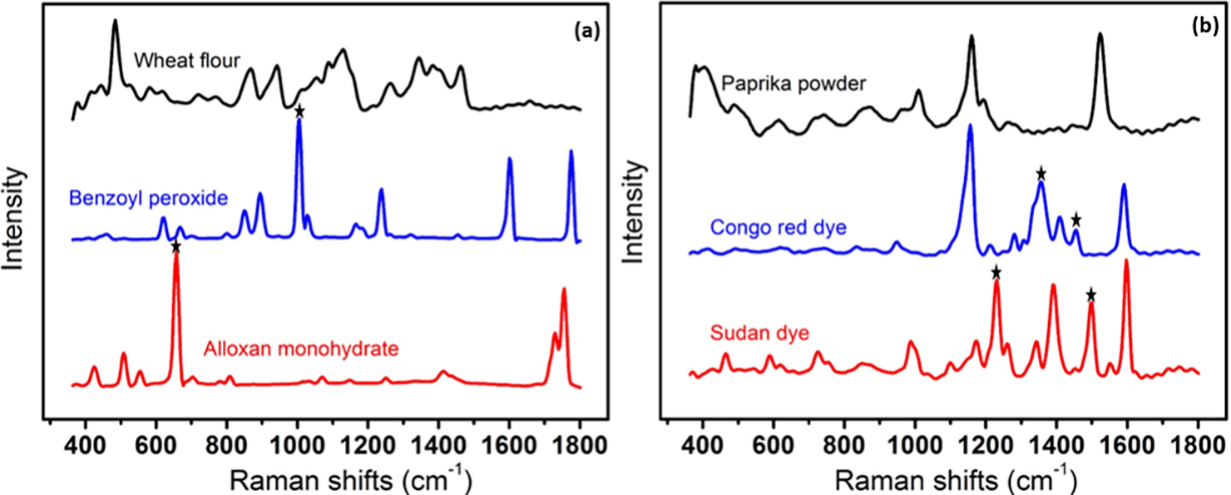
Fluorescence corrected Raman spectra of pure food powder (wheat flour (a), and paprika powder (b)) samples and adulterant materials.

For the Raman image data of the layered samples, the intensities of all pixels were plotted against the Raman shifts and the intensities of the unique characteristic peaks of the adulterant (base) materials were examined to estimate the effective penetration depth. Fig 4B shows the Raman spectrum of the pure BPO base material and the mean Raman spectra of wheat flour at four different thicknesses over the base material. For the Raman data of the mixed samples, the preprocessed 2D data were reshaped as 3D data, where the Raman intensity values were dependent on two spatial (*x* and *y*) and one spectral dimension. Therefore, each band has an intensity image of the whole scanned sample. Since the Raman spectra of BPO and alloxan monohydrate present fine and well resolved peaks (Fig 5A), and no interference by any notable peaks of the powdered food (i.e., wheat flour) was observed, a univariate method, which employs a single band intensity, was adopted to map these adulterant materials in wheat flour by plotting the frequencies of identified adulterant peak bands as a function of spatial position and spectral intensity. However, although the adulterant materials present in paprika powder (i.e., Sudan dye and Congo red) exhibited a number of well resolved peaks, a degree of interference was observed, as shown in Fig 5B. Therefore, the application of a univariate method in this case would likely lead to misclassification. To address this, a bivariate method was employed, which uses the sum of two different band intensities. Raman images of the two different bands at which Sudan dye exhibits high intensity peaks and peaks exhibiting the lowest interference from other adulterant materials or powdered food samples were added together to generate a band summation image to essentially enhance the pixels of the Sudan dye adulterant. This methodology was also applied to generate the summation image for the Congo red dye.

Finally, the single band images of the wheat flour adulterants and the summation images of the paprika powder adulterants were subjected to image segmentation to isolate the adulterant pixels from the powdered food background by selecting a threshold value. The binary image of the individual adulterant of the same sample was then combined to form a chemical image of the two identified adulterants. The concentration of each individual adulterant in the wheat flour and paprika powder samples was then calculated using the number of pixels belonging to that particular adulterant material in the binary image. The detected pixels for each adulterant at all concentration levels for both powdered food samples were correlated with the adulterant concentrations in the samples. All image correction and analysis processes were programmed in MATLAB (MathWorks, Natick, MA, USA).

## Results and discussion

### Penetration depth determination

As a demonstration of the developed RHSI system, we initially examined the layered powder samples. The penetration depth of the laser line through the wheat flour and paprika powder was measured following the method used in [29] to ensure that no adulterant particles at the bottom of the sample would be missed. The presence of signals corresponding to the base materials can be considered as a measure of the laser penetration depth when a univariate or bivariate method is of interest for data analysis. However, the whole spectral pattern and peak intensities should be considered as a measure of penetration depth when a multivariate method is of interest for further data analysis of the experimental samples.

A gradual decrease in the average spectral peak intensity of adulterant can be observed with increasing sample depth (Fig 4B). During evaluation of each pixel spectrum for the layered samples of different thicknesses, we observed that for a depth of 3 mm not all pixels were present in the peak of the base material, and only a few pixel spectra exhibited a peak at 998.7 cm^−1^ at a depth of 4 mm. Furthermore, the collected Raman image data for the layered paprika powder and Sudan dye samples were evaluated using the same method to ensure effective penetration depth determination. These results suggested that under the current laser powder and system setup, the maximum effective penetration depth of the line laser through the wheat flour and paprika powder samples is ~2 mm, and so this thickness will be employed for further (mixed sample) investigations.

### Raman image data correction

**Fig 6:**
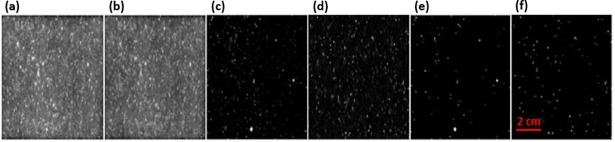
Raman images of 0.5% adulterated wheat flour: Images of selected wavebands for benzoyl peroxide (a) and alloxan monohydrate(b); (c) and (d) are preprocessed images of (a) and (b), respectively; (e) and (f) are binary images of (c) and (d), respectively.

The Raman band images of benzoyl peroxide and alloxan monohydrates are shown in Fig 6A and 6B, respectively. Despite representing different adulterants, both images (i.e., Fig 6A and 6B) exhibit similar features, and so no clear information can be determined regarding the distribution of the adulterants. This is due to the high intensity of the cosmic spikes and the fluorescence background from the wheat flour sample. Thus, to achieve further statistical analysis, the hyperspectral pixels of the collected Raman image data for both the wheat flour and paprika powder mixtures were initially fluorescence corrected using the airPLS method, prior to employing a median filter to remove the effect of cosmic spikes and to reduce noise. The corrected Raman images of the selected bands are visible at 998.7 and 653.3 cm^−1^ (Fig 6C and 6D), which correspond to the selected Raman shifts for BPO and alloxan monohydrate in wheat flour, respectively, and the corresponding binary images are given in Fig 6E and 6F, respectively. Removal of the fluorescence baseline and cosmic spikes thus enhances the Raman images and a relatively constant background can be seen in the corrected images. Visual inspection of the corrected images reveals the distribution of BPO and alloxan monohydrate particles as bright pixels. Fluorescence correction and removal of the cosmic spikes was also carried out to the Raman images of the adulterated paprika powder to yield similar results.

### Univariate and bivariate analysis

As mentioned above, the spectra of BPO and alloxan monohydrate exhibited the most intense bands at 998.7 and 653.3 cm^−1^ (Fig 5A), respectively. Comparison of the spectra shown in Fig 5A shows that these bands do not overlap, and are not significantly affected by any spectral features of the wheat flour sample in these regions. As such, the univariate images based on these bands can be considered for visualization of the presence and spatial distribution of these adulterants in wheat flour. By adopting a univariate method, which uses a single band intensity, a chemical map of each adulterant was created by plotting these unique wavebands as a function of spatial position and intensity. The univariate method is a straightforward method to visually determine the spatial distribution of a single chemical component. Importantly, this method uses only a small portion of data, and so the computation time is short (fraction of second), thereby rendering this method suitable for the online application of Raman imaging in the food quality analysis.

To quantify the individual adulterants present in the paprika powder sample, several options were examined. In an attempt to quantify Sudan dye and Congo red, the use of a univariate method based on the most intense peak for each adulterant resulted in a lower correlation coefficient for Sudan dye and false positive pixels for Congo red. We expect that this is due to the presence of interference from the adulterants and from the paprika powder itself, as indicated in Fig 5B. In addition, other spatially resolved bands were relatively weak in intensity, making it difficult to detect lower concentration levels. To address this issue and to effectively determine the adulterant concentrations, a bivariate analysis method was used. Bivariate analysis involves the use of two data points, such as two different band intensities, to create a band summation or a band ratio image. In this study, the summation of two bands, i.e., 1227 and 1493 cm^−1^ for Sudan dye and 1351 and 1451 cm^−1^ for Congo red, were chosen to enhance and segregate the adulterant particles from the paprika powder background and thus, to develop a quantitative model. The possible combination of other bands for both adulterants was also evaluated, but optimal results were achieved using the above combinations.

**Fig 7:**
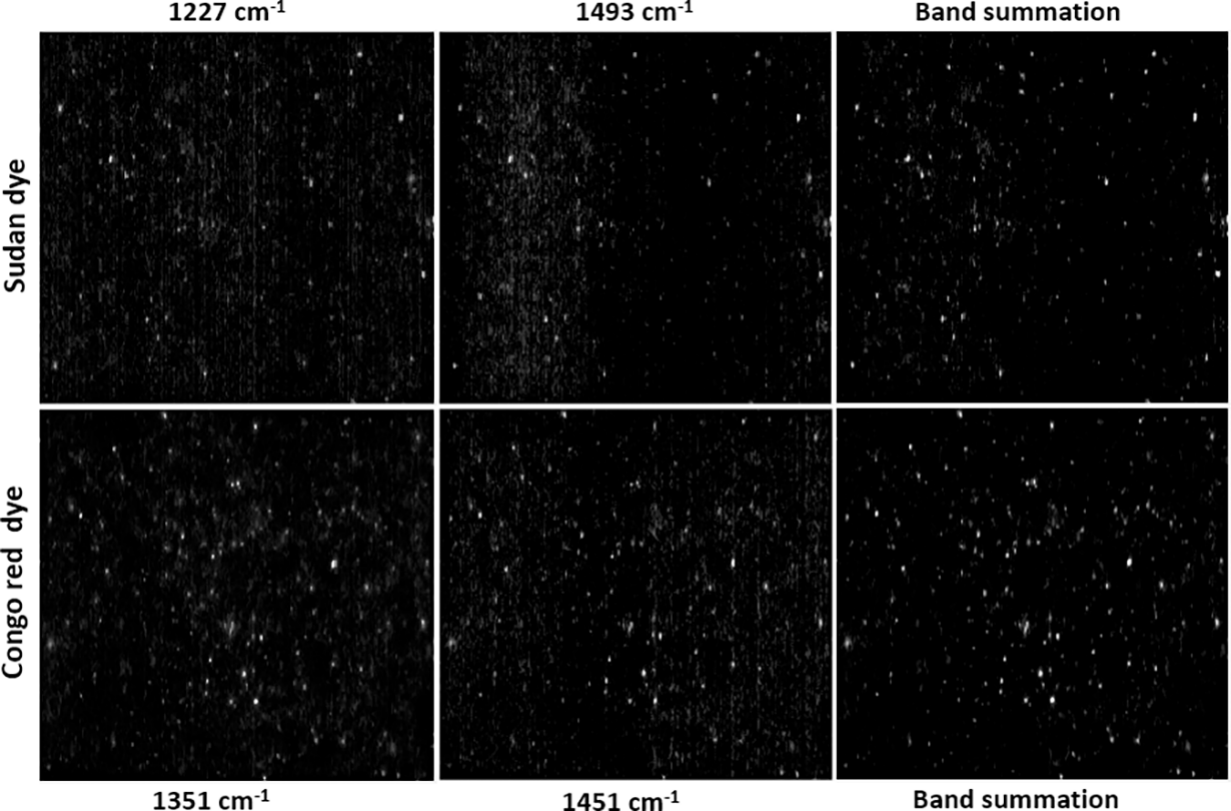
Preprocessed Raman images of a 1% adulterated paprika powder at selected bands of the adulterants and band summation images.

Fig 7 shows the preprocessed band images for Sudan dye and Congo red dye for the 1% adulterated paprika powder sample. As indicated, no difference in the spatial position of the high intensity (adulterant) pixels was observed in the two different band images of the same adulterant. However, due to the low intensities of the bands at 1493 cm^−1^ for Sudan dye and 1451 cm^−1^ for Congo red dye, it appears that the comparatively higher amount of background noise was caused by the rough texture of the paprika powder, as no such background noise was observed for the Raman band images of wheat flour. In addition, the band summation result (Fig 7) confirms the successful and effective segregation of adulterant pixels from the background noise through addition of the high intensity pixels at the same spatial positions corresponding to the adulterants. It could therefore be concluded that the bivariate method is effective for both adulterants, and a generated binary image gives improved results for adulterant quantification compared to a single band image.

### Quantification of the adulterants

To quantify the individual adulterant concentrations in two different powdered food samples and to ease the visualization of the spatial distribution of the adulterant materials, binary images were generated for each adulterant by applying threshold values to the intensities of the corrected images, ultimately generating false-color composite images. Selection of a suitable threshold value is of particular importance to reduce the occurrence of false positive and false negative pixels. In the case of wheat flour, the threshold value was determined based on the preprocessed intensity values (in the lowest and highest adulterant concentrated samples) at the two unique Raman peaks of the two adulterants. However, for quantification of the adulterant pixels in paprika powder, the threshold value was determined based on the band summation image of each dye. Each pixel counted as either adulterant or food powder was evaluated to avoid false-positive and false-negative determination.

**Fig 8:**
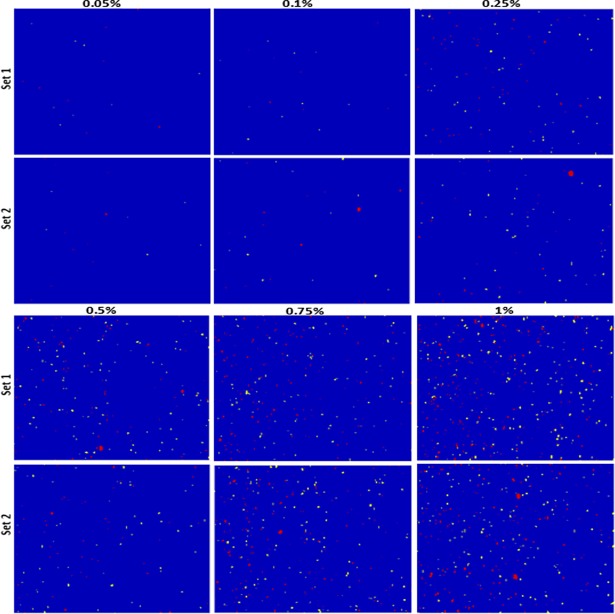
Combined color coded chemical images of benzoyl peroxide (red) and alloxan monohydrate (yellow) in wheat flour (blue background) with six different concentrations and two replications.

**Fig 9:**
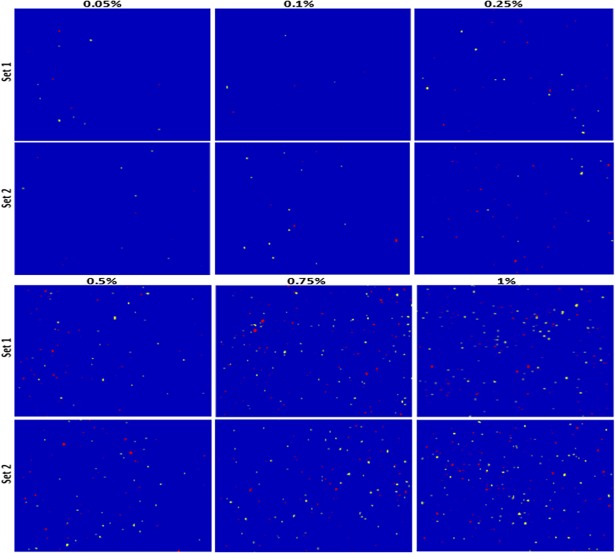
Combined color coded chemical images of Sudan dye (red) and Congo red dye (yellow) in paprika powder (blue background) with six different concentrations and two replications.

The obtained threshold values were then applied to convert all pixels with intensities below the threshold value into background pixels, while identifying all pixels with intensities above the threshold as representing a particle of the adulterant of interest. At each concentration level, the binary images of the two adulterants were combined and the pixels of each component were color-coded by assigning a particular value while thresholding. The final color-coded images for all six adulterant concentrations and two replications for both wheat flour and paprika powder are shown in Figs 8 and 9, respectively. In addition, the Raman mapping images do not only give information regarding the spatial distribution of the adulterants, but they also provide information regarding the particle size. Thus, from the color-coded images (Fig 8), the relatively larger size of the BPO particles compared to the alloxan monohydrate particles is apparent. However, no significant difference can be observed between the particle sizes of Sudan dye and Congo red (Fig 9).

**Fig 10:**
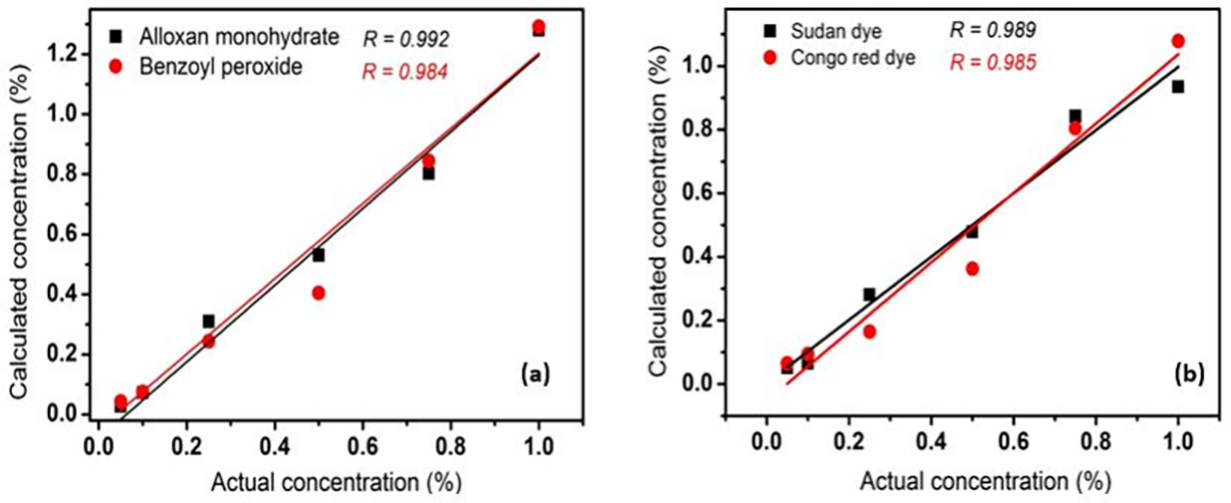
Linear relationship between the pixel based calculated concentrations corrected with the ratio of densities and added mass concentration of each adulterant in wheat flour (a) and paprika powder (b).

Once the binary images have been generated for the adulterants, the mass concentration of each individual adulterant can be calculated by calculating the percentage of adulterant pixels in the binary image. Thus, the pixel concentration was calculated for each adulterant in all measured samples, and an average was calculated using the replicated samples. As expected, the number of detected pixels increased considerably with increasing mass concentration of the adulterants, with a linear trend being observed for all adulterants in both powder samples. However, the pixel concentrations in the binary images were lower than the corresponding mass concentrations of the adulterants. We believe that this is caused by the significant difference in density between the adulterants and the food powders, as the density ratios between BPO and wheat flour and between alloxan monohydrate and wheat flour are approximately 1.5 and 1.65, respectively, while those between Sudan dye and paprika powder and between Congo red and paprika powder were approximately 2.4 and 2.16, respectively. These higher adulterant densities decrease their particle volumes when the mixed samples are prepared using the weight-by-weight concentrations. Therefore, to convert the pixel concentration into a mass concentration, each pixel concentration was multiplied by the corresponding density ratio, and the results are presented in Fig 10. Indeed, in this case, no significant variations between the calculated and added mass concentrations of the adulterant materials were observed, and a strong linear relationship was found with a correlation coefficient of >0.98 for all adulterants in both powdered samples. However, the small difference observed may be due to a number of factors mentioned in [23].

As the main objective of the present work was to develop and test the RHSI system for the authenticity analysis of powdered food, the result clearly demonstrates the capability of the RHSI technique to detect and spatially visualize the presence of adulterants in such samples. Although only two adulterants were investigated for each powdered food, we expect that the detection of multiple adulterants will be possible using a similar methodology or by adopting multivariate methods for data analysis when selective information from multiple spectral channels is not available.

Compared with previous attempts regarding authenticity analysis of wheat flour using Raman imaging [22,24], the current study demonstrated the similar results with the advancement of simultaneous detection of two potential adulterants. Moreover, no study has been utilized Raman imaging technique for rapid authenticity analysis of paprika powder which is one of the most vulnerable powdered food to be adulterated with hazardous chemical adulterants. In addition, the developed Raman imaging system along with the custom-build software seems to be more suitable to the industrial application because of the following reasons; firstly, unlike the previous system [20] where a moving mirror was used to generate the laser line, current system uses a specific arrangement of optics for the same purpose thus mechanically stable and can be operated continuously for relatively long time to suits the industrial environment. Secondly, the developed software facilitates the real-time visualization of Raman chemical images of adulterants, therefore, each single scan can be evaluated (while scanning) for the presence of adulterant representing the particular region of sample unlike the previous studies where Raman images of whole sample surface (in sample holder) were collected and analysed separately for the visualization of adulterant particle. Therefore, this distinct feature of developed system has advantage of speed because one can consider the sample as adulterated without measuring the whole sample if the adulterants’ pixels are visualized at early scans. Additionally, the developed RHSI system exhibits broad flexibility and can be employed for larger sample sizes by changing the objective lens and increasing the sample-to-lens distance if a larger pixel size is of interest. Moreover, Raman microscopic imaging has been employed in pharmaceutical quality analysis, including in the chemical characterization and quantitative information prediction of pharmaceutical end products [30]. We therefore expect that our macroscopic-scale RHSI system has potential for application in the rapid quality analysis of pharmaceutical products in areas where microscopic-level resolution is not necessary.

## Conclusion

We herein described the development and calibration of a rapid line-scan Raman hyperspectral imaging technique (RHSI) technique with custom-built software and its use in the detection of food adulteration and authenticity analysis of two different powdered food samples. Following the collection of Raman images for the layered samples to determine the effective penetration depth of the laser (optimal depth = 2 mm), mixed (adulterated) powder samples were examined. The quantification of individual adulterants in adulterated powder was possible through the application of a threshold value to the intensity value of the corrected band images or summation images (at all concentration levels) for the adulterant of interest. Indeed, a strong linear relationship (*R*>0.98) was found between the mass concentration and the pixel-based detected concentration of adulterants in both wheat flour and paprika powder.

The obtained results therefore suggest that combination of this RHSI system with univariate and bivariate data analysis techniques is a rapid and effective tool for the detection of chemical adulteration in food powders. We expect that this study can easily be extended to simultaneously detect various adulterants in different kinds of food powders even on a large scale. In addition, the proposed methodology will provide a novel approach to the rapid determination of food authenticity and can also be considered for pharmaceutical quality analysis. We therefore expect that it exhibits potential for industrialization in the near future. Future studies will focus on the application of our system for the detection of multiple possible adulterants in adulteration-susceptible powdered foods, in addition to the quality analysis of dairy products, fruits, and vegetables.
